# Examining the Relationship between Economic Hardship and Child Maltreatment Using Data from the Ontario Incidence Study of Reported Child Abuse and Neglect-2013 (OIS-2013)

**DOI:** 10.3390/bs7010006

**Published:** 2017-02-08

**Authors:** Rachael Lefebvre, Barbara Fallon, Melissa Van Wert, Joanne Filippelli

**Affiliations:** 1 Factor-Inwentash Faculty of Social Work, University of Toronto, 246 Bloor Street West, Toronto, ON M5S 1V4, Canada; barbara.fallon@utoronto.ca (B.F.); joanne.filippelli@mail.utoronto.ca (J.F.); 2 Centre for Research on Children and Families, McGill University, 3506 University Street, Montreal, QC H3A 2A7, Canada; melissa.vanwert@utoronto.ca

**Keywords:** child maltreatment, economic hardship, developmental outcomes, resilience

## Abstract

There is strong evidence that poverty and economic disadvantage are associated with child maltreatment; however, research in this area is underdeveloped in Canada. The purpose of this paper is to examine the relationship between economic hardship and maltreatment for families and children identified to the Ontario child protection system for a maltreatment concern. Secondary analyses of the Ontario Incidence Study of Reported Child Abuse and Neglect-2013 (OIS-2013) were conducted. The OIS-2013 examines the incidence of reported maltreatment and the characteristics of children and families investigated by child welfare authorities in Ontario in 2013. Descriptive and bivariate chi-square analyses were conducted in addition to a logistic regression predicting the substantiation of maltreatment. In 9% of investigations, the household had run out of money for food, housing, and/or utilities in the past 6 months. Children in these households were more likely to have developmental concerns, academic difficulties, and caregivers with mental health concerns and substance use issues. Controlling for key clinical and case characteristics, children living in families facing economic hardship were almost 2 times more likely to be involved in a substantiated maltreatment investigation (OR = 1.91, *p* < 0.001). The implications in regard to future research and promoting resilience are discussed.

## 1. Introduction

It has been well documented that children living in poverty experience a wide range of disadvantages in the areas of physical and mental health, development, and academic achievement [1,2,3,4]. In Canada, several cohort studies have shown that children living in poverty are more likely to develop health problems [5], to display disruptive behavior [6], and to drop out of high school [7]. Research has also identified that experiencing poverty at both the individual as well as at the neighborhood level is associated with serious externalizing and internalizing behavior problems in children [8]. Furthermore, poverty and economic disadvantage have long been associated with a greater risk of child maltreatment [9,10,11]. In the United States, children living in financially strained households are at five times greater risk for child abuse and neglect compared to children from families with higher socio-economic status [12]. Neglect is the most frequently investigated type of maltreatment in the United States [13] and also the type most commonly associated with poverty [11,13,14].

Risk factors that are associated with an increased risk of poverty are also associated with an increased risk of child maltreatment; therefore, disentangling the causal role or mechanisms for the observed associations between poverty and child maltreatment is challenging [15]. Various hypotheses have been forwarded [15,16,17,18,19]. Lower socio-economic status is theorized to be a significant risk factor for child maltreatment because of the stress it places on the caregivers in the family and its relationship to social supports and access to resources [16]. Economic hardship could also adversely impact parenting quality and capacity through changes in parental mental health, parenting behaviors, or family dynamics [17,18,20]. Families facing economic disadvantage may also struggle to financially meet the basic needs of their families [15].

It has also been hypothesized that indicators of poverty may act to increase the visibility or scrutiny of low income families with respect to official reports of child maltreatment [15]. Children living in poor families are more likely to have child welfare involvement than those who are not poor [19,21,22], and child welfare system involvement has also been associated with indicators of socio-economic disadvantage, including challenges in paying for housing, food, and utilities [15]. Alternatively, poor families may be more likely to be reported for incidents of maltreatment because of the greater array of risk factors and stressors they experience and not as a result of higher levels of scrutiny or class bias relating to reporting [19]. For example, reported children from lowincome families were four times more likely to have a parent with an identified mental health issue [19]. While economic hardship is associated with a higher likelihood of maltreatment, financial support can potentially prevent abuse and neglect. The findings of a recent longitudinal random assignment experiment indicate that income support to families can play a causal role in reducing the likelihood of child maltreatment [15], and another recent study found that increasing the minimum wage by $1 led to significantly fewer reports of child neglect [23].

Whether or not child maltreatment occurs within a family system depends on the balance between risk factors and protective factors, of which economic disadvantage is just one risk factor [24,25,26]. Epidemiological studies underscore that the age of children influences the risk of maltreatment [27,28]. For instance, infants are more likely to be investigated [29] and be substantiated for maltreatment [30]. Research suggests that families with younger children also tend to experience greater socio-economic hardships than those with older children [29,31]. Parental factors such as young caregiver age, substance abuse, and mental health concerns are also established as risk factors for child maltreatment [32,33,34,35].

Child maltreatment can have long-term consequences for children’s physical and mental health, substance misuse, and other risk behaviors [36,37,38,39]. Children living in families facing economic hardship who are also involved with child welfare due to confirmed or suspected maltreatment may represent a particularly vulnerable group and therefore warrant further study. Research in the area of economic hardship and child maltreatment is underdeveloped in Canada compared to the United States [40]. We do not know basic information about those families struggling with economic disadvantage who also come to the attention of the child welfare system [40]. The Canadian context is unique, with universal health care and a more extensive network of social welfare programming [41]. Rates of reported neglect are considerably lower in Canada than in the United States [42] and the rate of child poverty is also lower in Canada. Despite this lower rate of poverty, Canada was recently ranked only 24th of 35 industrialized countries for relative child poverty [43], and the most recent iteration of the General Social Survey found that just under a quarter (23%) of Canadian households were ‘unable to make ends meet’ [44]; therefore poverty and economic hardship remain a significant concern in Canada.

This paper explores the relationship between economic hardship and maltreatment using data from the 2013 cycle of the Ontario Incidence Study of Reported Child Abuse and Neglect (OIS-2013). The main objectives of this paper are to: (1) obtain a profile of economic hardship in a representative Provincial sample of child welfare investigations; and (2) investigate whether economic hardship is associated with key child, family, and case characteristics, including the substantiation of maltreatment (physical abuse, sexual abuse, neglect, emotional maltreatment, and exposure to intimate partner violence; please see Table 1 for definitions). The following four research questions are addressed:

(1) How many children investigated by child welfare authorities in Ontario live in families facing economic hardship?
(2) What are the characteristics of children who live in families facing economic hardship and are referred to a child welfare service for a maltreatment-related concern?
(3) Are children who live in families facing economic hardship more likely to be involved in a substantiated maltreatment investigation, controlling for key clinical and case characteristics?
(4) Do children who live in families facing economic hardship experience unique forms of maltreatment?

Given prior research findings, we hypothesized that we would find a relationship between economic hardship and the decision to substantiate child maltreatment, whereby a noted concern of economic hardship would increase the likelihood of maltreatment substantiation. Greater understanding of this intersection will assist in the development of efficacious interventions which can attend to the immediate concerns of families in addition to addressing the safety of children while promoting positive cognitive, emotional, and developmental outcomes.

## 2. Materials and Methods

Secondary analyses of data collected in the 2013 Ontario Incidence Study of Reported Child Abuse and Neglect (OIS-2013) were conducted to meet the objectives of this paper [45] The OIS-2013 is the fifth provincial study to examine the incidence of child maltreatment in Ontario. Ontario is the most populated province in Canada, and just under 40% of the Canadian population lived in Ontario in 2013 [46]. The OIS-2013 captures information on investigation outcomes, forms and severity of maltreatment, and the characteristics of children and families investigated by child welfare authorities in Ontario [45]. Using a multi-stage sampling design, a representative sample of 17 child welfare sites was first selected from 46 child welfare organizations in Ontario. Then cases opened between a three month period from 1 October 2013 to 31 December 2013 within these selected sites were sampled for inclusion [45]. Maltreatment-related investigations that met the criteria for inclusion in the OIS-2013 included situations in which there were concerns that a child may have already been abused or neglected (maltreatment investigations) as well as situations in which there was no specific concern about past maltreatment but where the risk of future maltreatment was being assessed (risk investigations). These procedures yielded a final sample of 5265 child maltreatment-related investigations [45]. Weighted national annual estimates were derived based on these investigations. Please see Fallon et al. (2015) [45] for a detailed description of weighting procedures.

Data was collected directly from investigating child welfare workers upon the completion of their initial investigation. The OIS-2013 had an item completion rate of over 99% for all items [45]. For several questions, the worker could identify a response option of ‘unknown’. For the present analysis we treated ‘unknowns’ as missing data and excluded investigations where ‘unknown’ was indicated for the socio-economic variables included in this analysis. While empirically investigations with ‘unknown’ responses on the socio-economic variables were not statistically different than investigations with no concerns for these issues, we did not include these investigations in order to be more conservative in our analysis and hence our knowledge claim. Moreover, community caregiver investigations were also excluded from this analysis (*n* = 72). A community caregiver is defined as anyone providing care to a child in an out-of-home setting (e.g., institutional setting). In these investigations, family information was not collected as it was not applicable. The present analyses were therefore based on an unweighted sample of 3790 maltreatment-related investigations with full information on the variables of interest.

There were several items collected in the OIS-2013 that are intended to measure the socio-economic conditions of the household. These items reflect the information that a worker would know at the end of their initial investigation. The frequencies of these socio-economic conditions are presented in Table 2 in the results section below. In order to assess whether the family was able to meet basic necessities, workers were asked to identify whether the household had run out of money for food, housing, and/or utilities in the past six months. Our composite measure of economic hardship is derived by noting whether a household was noted by the worker as experiencing any one of these conditions.

Descriptive and bivariate chi-square analyses were conducted on the weighted sample of maltreatment-related investigations to determine the characteristics of children living in families facing economic hardship, based on the variables available in the OIS-2013 study. Chi-square tests of significance were conducted using the sample weight, which adjusts for the inflation of the chi-square statistic by the size of the estimate by weighing the estimate down to the original sample size. A logistic regression was conducted on the unweighted sample of 2967 maltreatment investigations to examine the relationship of poverty to the decision to substantiate child maltreatment, with seven cases having been excluded list wise. Unweighted data were used in this multivariate analysis to ensure unbiased results due to the inflation of significance due to a large sample size. The predictors were entered into a multiple logistic regression in four blocks to represent an ecological model of maltreatment, with child factors entered into the first block, caregiver factors entered into the second block, case characteristics entered into the third block, and our variable of research interest, economic hardship, in the fourth (last) block along with our other socio-economic variables. Finally, bivariate chi-square analyses were completed on substantiated maltreatment investigations to explore the relationship between economic hardship and characteristics of substantiated maltreatment, including subtypes of neglect. Please see Table 1 for a complete description of the variables used in these analyses. A complete OIS-2013 guidebook is available in Fallon et al., (2015) [45].

## 3. Results

As shown in Table 2, in over a quarter of maltreatment-related investigations, the family relied on social assistance or other government benefits as their main source of income. In the majority of investigations, the family did not own their own home and in six percent of investigations, the family was living in a temporary arrangement (e.g., hotel, shelter, living with friends/family). In seven percent of investigations, the worker indicated there was home overcrowding, and, in five percent of investigations, the worker noted unsafe housing conditions. In just under ten percent of investigations, the worker indicated that the household had run out of money for food, housing, and/or utilities. When we use the term economic hardship throughout the rest of this paper, we are referring to this measure: insufficient income to meet basic necessities.

Table 3 compares several case characteristics for those investigations in which the child was living in a family facing economic hardship compared to those investigations in which the worker did not note economic hardship as a concern. All of the primary caregiver risk factors were noted more frequently within investigations where the child was living in a family facing economic hardship. Few social supports and mental health issues were more than two times as likely to be noted in investigations in which economic hardship was a concern, and alcohol/substance abuse issues were almost four times are likely to be noted. Children living in a family facing economic hardship were more likely to have had two or more previous child welfare investigations (65% vs. 44% of investigations) and less likely to have had no previous child welfare involvement (21% vs. 38%). Developmental concerns and academic difficulties for children were noted more frequently in investigations in which the worker noted economic hardship as a concern. However, there were no statistically significant differences in the prevalence of externalizing and internalizing functioning concerns between children who were living in a family facing economic hardship and children who were not.

Table 4 presents the results of the multiple logistic regression, which was conducted to examine whether substantiation of child maltreatment is significantly predicted by whether the family experiences economic hardship, controlling for child age and ethnicity, caregiver age and functioning concerns (i.e. substance abuse, mental and physical health issues), maltreatment type, previous substantiated maltreatment to the child, and other indicators of socio-economic disadvantage. The goodness of fit of the overall model is good as it correctly classifies 70.4% of the total sample and the chi-square of the model (*x*^2^ = 718.876) is significant (*p* < 0.001).

Our analysis indicates that caregiver substance abuse, caregiver mental and physical health issues, type of maltreatment, previous substantiated maltreatment, home overcrowding, unsafe housing, and economic hardship are significant predictors of the substantiation of current maltreatment as each Odds Ratio (OR) is significant. For our research variable of interest, economic hardship, the OR is 1.91 (*p* < 0.001), meaning that if the family faces economic hardship, the odds of the child having a substantiated maltreatment concern increase by a factor of 1.91. After controlling for the other relevant predictors in our model, children in families facing economic hardship are almost 2 times more likely to be involved in a substantiated maltreatment investigation compared to those children not living in families facing economic hardship. A substantiated investigation indicates that the child was a victim of physical abuse, sexual abuse, neglect, emotional maltreatment, or exposure to intimate partner violence (IPV).

Table 5 outlines the characteristics of maltreatment for those substantiated maltreatment investigations in which the worker noted the child was living in a family facing economic hardship compared to those substantiated maltreatment investigations in which economic hardship was not a concern. Children living in a family facing economic hardship were significantly more likely to have a primary substantiated maltreatment type of neglect (43% vs. 21% of investigations). With regard to the duration of maltreatment, the presence of mental/emotional harm, and physical harm to the child, there were no significant differences between investigations with a noted concern of economic hardship compared to investigations without a concern of economic hardship.

Table 6 presents the specific subtypes of neglect for those substantiated maltreatment investigations in which the child was living in a family facing economic hardship compared to those investigations in which economic hardship was not a noted concern. Children living in a family facing economic hardship were most likely to be involved in a substantiated physical neglect investigation, whereas, in investigations without a concern of economic hardship, children were most likely to be involved in a substantiated failure to supervise investigation. Physical neglect was defined as a child who suffered or was at substantial risk of suffering physical harm caused by the caregiver(s)’ failure to care and provide for the child adequately, which included inadequate nutrition/clothing and unhygienic dangerous living conditions.

## 4. Discussion

This paper has shown that a significant proportion of children investigated by child welfare authorities in Ontario live in families struggling with economic hardship, and the children who live in these families are more likely to have developmental concerns and academic difficulties along with high-risk caregivers and previous child welfare involvement. Our results indicate that when controlling for demographics, caregiver risk, previous substantiated maltreatment, type of current maltreatment, and certain socio-economic variables, children living in families facing economic hardship are significantly more likely to be victims of maltreatment. Children who live in families struggling with economic hardship are more likely to experience neglect, specifically physical neglect. While the social welfare programming in Canada is more extensive than in the United States [41], we see a similar relationship between economic hardship and maltreatment, specifically neglect. Our findings emphasize that families identified to child welfare who run out of money for basic necessities have multiple complex needs.

It is of utmost importance for the child welfare sector to consider how to best promote positive child adaptation in the context of such multiple adversities. A child’s relationship with at least one stable, caring, responsive, and supportive adult has been noted as the most critical developmental protective factor for promoting resilience [47,48]. The circumstances and stress associated with lacking money to pay for basic necessities can detrimentally impact a caregiver’s ability to engage in positive parenting behaviors [49]; therefore, focusing on building positive child-caregiver relationships among children who live in families facing economic hardship is critical.

For those families facing economic hardship who are identified to child welfare, the child welfare system can be viewed as part of the environmental context that influences children, their caregivers, the child-caregiver relationship, and, ultimately, children’s developmental resilience. Unfortunately, there is a dearth of research that focuses on social services as facilitators of positive adaptation in the context of child maltreatment [50], and there is a lack of longitudinal studies that aim to understand the dynamic qualities of resilience following maltreatment [50,51]. Additional research is needed to understand the children and families that experience socio-economic hardship that come to the attention of the child welfare system. In order to identify efficacious interventions for these families, research should ultimately focus on understanding the unique factors that promote resilience among this particularly vulnerable subgroup of children. Resilience research is necessary to inform theory and practice across multiple levels of analysis that may include factors at the individual, family, and community levels [52,53].

It is also likely for resilience to be enhanced through attention to national economic policies that reduce the prevalence of children and families facing economic hardship. Since the child welfare system has a limited capacity to address systemic issues of economic disadvantage through casework, child welfare professionals should act as advocates for these broad policy level interventions. These investments may even result in cost savings to the child welfare system, a system that utilizes almost $1.5 billion in public funds each year in Ontario [54], by mitigating the need for a child protection response.

### Study Limitations

The OIS is a cross sectional study and therefore cannot provide causal evidence for the relationship between economic hardship and child maltreatment. The OIS does not include incidents of unreported maltreatment nor does it include cases that were investigated only by the police. Also, reports that were screened out by child welfare authorities (not opened for investigation) were not included. Similarly, reports on cases currently open at the time of case selection were not included. The OIS collects information directly from child welfare workers at the point when they completed their initial investigation of a report of possible child abuse or neglect or a risk of future maltreatment [29]. Therefore, the scope of the study is limited to the type of information available to them at that point, and the study did not track longer-term service events that occurred beyond the initial investigation. Moreover, the information collected was from the workers’ clinical perspective and was not independently verified. The OIS child functioning checklist included within the study’s standardized data collection instrument is not a validated measurement instrument with established population norms for child functioning concerns [29].

Three limitations to the weighting estimation method should be noted. The agency size correction uses child population as a proxy for agency size; this does not account for variations in per capita investigation rates across agencies in the same strata. The annualization weight corrects for seasonal fluctuation in the volume of investigations, but it does not correct for seasonal variations in the types of investigations conducted. Finally, the annualization weight includes cases that were investigated more than once in the year as a result of the case being re-opened following a first investigation completed earlier in the same year. Accordingly, the weighted annual estimates represent the child maltreatment-related investigations, rather than the investigated children.

## 5. Conclusions

Our findings indicate that children experiencing economic disadvantage were more likely to have developmental concerns and academic difficulties and to experience victimization. These findings are a stark reminder of the opportunity and need to focus prevention, intervention, and research efforts on children who are dealing with the burdens of economic hardship and maltreatment, two well-established and salient developmental risk factors. The vulnerability of children highlighted in this study underscores the need for accessible and responsive services that promote resilience and mitigate the impact of adversity within the child welfare sector in partnership with other allied service sectors (e.g., children’s mental health, adult mental health, education). It is also evident that the policy context needs to be examined and considered in order to ensure investments are made in prevention-focused economic redistribution policies that can support positive child developmental outcomes.

## Figures and Tables

**Table 1:** Variables used in the present analyses.

Variable	Definition
Child Age	Workers were asked to indicate the age of the investigated child.
Child Ethno-racial Group	Workers were asked to indicate the ethno-racial background that best described the investigated child.
Child Functioning Concerns	Workers were asked to identify the child’s level of functioning for 18 concerns. For each functioning concern, response options were confirmed, suspected, no, or unknown. Confirmed and suspected responses were collapsed into ‘noted’, and no and unknown response options were collapsed into ‘not noted’. The following child functioning concerns were examined as part of this analysis: internalizing behaviors, externalizing behaviors, developmental concerns, and academic difficulties. Workers could note multiple child functioning concerns. Internalizing behaviors was derived by collapsing the following concerns: depression/anxiety/withdrawal, suicidal thoughts, self-harming behavior, and attachment issues. Externalizing behaviors was derived by collapsing the following concerns: ADD/ADHD, aggression, running from home, and Youth Criminal Justice Act involvement. Developmental concerns were derived by collapsing the following concerns: intellectual/developmental disability, failure to meet developmental milestones, and FAS/FAE (fetal alcohol syndrome/fetal alcohol effects).
Previous Substantiated Investigation	Workers were asked to indicate whether the investigated child had any alleged maltreatment against them substantiated prior to the current investigation.
Primary Caregiver Age	Workers were asked to indicate the age of the primary caregiver.
Primary Caregiver Risk Factors	Workers were asked to identify risk factors for the primary caregiver. For each risk factor, response options were confirmed, suspected, no, or unknown. Confirmed and suspected responses were collapsed into ‘noted’ and no and unknown response options were collapsed into ‘not noted’. This analysis examined the following caregiver risk factors: alcohol abuse, drug/solvent abuse, cognitive impairment, mental health issues, physical health issues, few social supports, and history of foster care or group home. Workers could note multiple risk factors. Two categories were derived for the purpose of this analysis: (i) alcohol abuse and drug/solvent abuse were collapsed for the bivariate and multivariate analyses; (ii) mental health issues and physical health issues were collapsed for the multivariate analyses.
Case Previously Opened	Workers were asked to indicate if the family had a case opened for child welfare services in the past, and could note that the case had never been previously opened, opened once before, opened two or three times before, opened more than three times before, or that they did not know.
Household Ran Out of Money for Food, Housing and/or Utilities	Workers were asked to indicate if the household had run out of money for food, housing, and/or utilities in the last six months. From these variables, we derived our composite measure of economic hardship by noting whether a household was noted by the worker as experiencing any one of these conditions.
Primary Maltreatment Category	Workers could identify up to three forms of investigated maltreatment from a list of 32 codes. For the primary maltreatment, workers were asked to indicate the maltreatment code that best characterized the investigation. These 32 codes were collapsed into five major maltreatment types: physical abuse (i.e. shake, push, grab, or throw; hit with hand; punch, kick or bite; hit with object; choking; poisoning; stabbing; or other physical abuse), sexual abuse (i.e. penetration; attempted penetration; oral sex; fondling; sex talk or images; voyeurism; exhibitionism; exploitation; or other sexual abuse), neglect (i.e. failure to supervise: physical harm; failure to supervise: sexual abuse; permitting criminal behavior; physical neglect; medical neglect; failure to provide psychological treatment; abandonment; or educational neglect), emotional maltreatment (i.e. terrorizing or threat of violence; verbal abuse or belittling; isolation/confinement; inadequate nurturing or affection; or exploiting or corrupting behavior), and exposure to intimate partner violence (IPV) (i.e. direct witness to physical violence; indirect exposure to physical violence; exposure to emotional violence; or exposure to non-partner physical violence). For more information on the definitions given to workers for each of the 32 forms of maltreatment, please see the OIS-2013 Guidebook in Fallon et al. (2015) [45].
Maltreatment Substantiation	For each form of maltreatment, workers were asked to indicate, based on their clinical judgment, the substantiation level for the investigation: unfounded (balance of evidence implied that the maltreatment did not occur); suspected (that there was not enough evidence to confirm that maltreatment had occurred, but maltreatment could not be ruled out); or substantiated (balance of evidence implied that the maltreatment occurred). Bivariate chi-square analyses were conducted to examine the similarity between suspected and substantiated cases. Since case characteristics of suspected cases were not significantly different from substantiated cases, we collapsed suspected and substantiated investigations for the logistic regression analysis.
Duration of Maltreatment	Workers were asked to indicate the duration of substantiated maltreatment as either a single incident or multiple incidents.
Household Income	Workers were asked to identify the primary source of income for up to two caregivers in the household. From these responses, a household income measure was derived.
Housing	Workers were asked to indicate the housing category that best described the living situation of the household at the time of the referral for investigation.
Home overcrowding	Workers were asked to indicate whether they felt, in their clinical opinion, that the household was overcrowded.
Unsafe housing	Workers were asked to identify if there were unsafe housing conditions (e.g., mold, inadequate heating, fire/electrical hazards).
Mental or emotional harm	When maltreatment was substantiated or suspected, the worker was asked to indicate whether the child was showing signs of mental or emotional harm.
Physical harm	Workers were asked to indicate if there was physical harm to the child.

**Table 2:** Percentage of socio-economic risk factors for all maltreatment-related investigations.

Socio-Economic Variables	#	%
Income		
Full-time	54,544	63
Part-time/seasonal	7776	9
Social assistance/benefits	22,663	26
None	1644	2
Housing type		
Own home	39,013	45
Rental	33,989	39
Public or band housing	8427	10
Temporary arrangement/other	5197	6
Home overcrowded	6453	7
Unsafe housing	3949	5
**Ran out of money for food, housing, and/or utilities**	**7710**	**9**
**Total**	**86,628**	**100**

Note: This bolded variable is the variable used for our bivariate and multivariate analyses to follow.

**Table 3:** Bivariate analyses for all maltreatment-related investigations.

Case Characteristics	Household Ran Out of Money				
	Yes		No		
	#	%	#	%	x^2^
Primary caregiver risk factors					
Alcohol/substance abuse	3107	40	8292	11	139.071 ***
Cognitive impairment	976	13	2535	3	67.288 ***
Mental health issues	3469	45	14,287	18	131.737 ***
Physical health issues	1244	16	4148	5	59.143 ***
Few social supports	3860	50	16,247	21	143.245 ***
History of foster care/group home	1188	15	2843	4	93.514 ***
Case history					
No previous investigation	1591	21	29,693	38	53.793 ***
One previous investigation	1118	14	14,348	18	
Two + previous investigations	5002	65	34,876	44	
Child functioning					
Internalizing issues	2335	30	22,284	28	1.219
Externalizing issues	2398	31	20,464	26	3.057
Developmental concerns	1902	25	13,478	17	15.512 ***
Academic difficulties	1793	23	13,018	16	9.219 **
**Total**	**7711**	**100**	**78,917**	**100**	

** *p* <0.01; *** *p* < 0.001.

**Table 4:** Multiple logistic regression predicting substantiation of maltreatment in maltreatment investigations.

Level	Variables Entering Model	*B*	*SE*	*p*-Value	Odds Ratio	95% Confidence Interval	
1	Child age	0.02	0.01	0.093	1.02	0.99	1.04
	Child ethnicity (white as reference)						
	Black	0.37	0.16	0.027	1.44	1.04	1.99
	Aboriginal	0.04	0.14	0.761	1.05	0.79	1.39
	Other	0.14	0.12	0.265	1.15	0.90	1.46
2	Young caregiver (over 30 years as reference)						
	21 and under	0.01	0.25	0.985	1.01	0.62	1.64
	22–30	−0.05	0.12	0.653	0.95	0.76	1.19
	Caregiver substance abuse	0.69	0.15	0.000	1.99	1.50	2.66
	Caregiver mental/physical health concern	0.55	0.10	0.000	1.74	1.42	2.13
3	Primary maltreatment type (physical abuse as reference)						
	Sexual abuse	−0.08	0.25	0.727	0.92	0.57	1.49
	Neglect	0.26	0.12	0.033	1.29	1.02	1.63
	Emotional maltreatment	1.23	0.15	0.000	3.43	2.56	4.59
	Exposure to IPV	2.00	0.12	0.000	7.39	5.85	9.32
	Previous substantiated maltreatment	0.66	0.09	0.000	1.98	1.65	2.38
4	Household income (full time as reference)						
	Part-time/seasonal	0.032	0.154	0.836	1.032	0.764	1.395
	Social assistance/benefits	−0.126	0.113	0.266	0.882	0.707	1.101
	None	0.20	0.32	0.531	1.22	0.66	2.26
	Housing type (own home as reference)						
	Rental	−0.04	0.10	0.681	0.96	0.78	1.18
	Public or band housing	−0.22	0.16	0.180	0.81	0.59	1.11
	Temporary arrangement/other	0.32	0.22	0.141	1.38	0.90	2.13
	Home overcrowding	0.49	0.18	0.007	1.62	1.14	2.31
	Unsafe housing conditions	1.30	0.23	0.000	3.63	2.32	5.70
	Household ran out of money	0.65	0.17	0.000	1.91	1.38	2.64

**Table 5:** Bivariate analyses for all substantiated maltreatment investigations.

Characteristics of Maltreatment	Household Ran Out of Money				
	Yes		No		
	#	%	#	%	x^2^
Primary maltreatment type					47.051 ***
Physical abuse	212	5	3359	13	
Sexual abuse	-	-	432	2	
Neglect	1716	43	5367	21	
Emotional maltreatment	273	7	4020	15	
Exposure to IPV	1791	45	12,812	49	
Duration					2.274
Single incident	1414	36	10,772	41	
Multiple incidents	2568	64	15,216	59	
Mental or emotional harm	1206	30	9498	37	2.606
Physical harm	110	3	1448	6	2.037
**Total**	**3982**	**100**	**25,988**	**100**	

*** *p* < 0.001.

**Table 6:** Bivariate analysis for subtypes of substantiated neglect

Subtypes of Neglect	Household Ran Out of Money				
	Yes		No		
	#	%	#	%	x^2^
Types of neglect					37.519 ***
Failure to supervise	605	35%	3148	59%	
Permitting criminal behaviour	-	-	-	-	
Physical neglect	730	43%	774	14%	
Medical neglect	202	12%	239	4%	
Failure to provide psych treatment	-	-	237	4%	
Abandonment	-	-	607	11%	
Educational neglect	126	7%	305	6%	
**Total**	**1716**	**100%**	**5367**	**100%**	

*** *p* < 0.001.
